# Management and Outcomes of Blunt Renal Trauma: A Retrospective Analysis from a High-Volume Urban Emergency Department

**DOI:** 10.3390/jcm14155288

**Published:** 2025-07-26

**Authors:** Bruno Cirillo, Giulia Duranti, Roberto Cirocchi, Francesca Comotti, Martina Zambon, Paolo Sapienza, Matteo Matteucci, Andrea Mingoli, Sara Giovampietro, Gioia Brachini

**Affiliations:** 1 Department of Surgery, Sapienza University of Rome, Viale del Policlinico 155, 00161 Rome, Italy; giuliaduranti95@gmail.com (G.D.); comotti.francesca22@gmail.com (F.C.); paolo.sapienza@uniroma1.it (P.S.); andrea.mingoli@uniroma1.it (A.M.); gioia.brachini@uniroma1.it (G.B.); 2 Department of Surgery, General Surgery, University of Perugia, 06129 Perugia, Italy; roberto.cirocchi@unipg.it; 3 Department of Surgery, Ospedale Santa Rosa, 01100 Viterbo, Italy; martina.zambon@uniroma1.it; 4 Department of Surgery, University of Milan, 20122 Milan, Italy; matteo.matteucci@unimi.it; 5 Department of Surgery, ICOT Istituto Marco Pasquali, 04351 Latina, Italy; saragiova91@gmail.com

**Keywords:** blunt renal trauma, AAST classification, non-operative management, nephrectomy, shock index, active bleeding, ISS, mortality

## Abstract

**Background:** Renal trauma accounts for approximately 3–5% of all trauma cases, predominantly affecting young males. The most common etiology is blunt trauma, particularly due to road traffic accidents, and it frequently occurs as part of polytrauma involving multiple organ systems. Management strategies are primarily dictated by hemodynamic stability, overall clinical condition, comorbidities, and injury severity graded according to the AAST classification. This study aimed to evaluate the effectiveness of non-operative management (NOM) in high-grade renal trauma (AAST grades III–V), beyond its established role in low-grade injuries (grades I–II). Secondary endpoints included the identification of independent prognostic factors for NOM failure and in-hospital mortality. **Methods:** We conducted a retrospective observational study including patients diagnosed with blunt renal trauma who presented to the Emergency Department of Policlinico Umberto I in Rome between 1 January 2013 and 30 April 2024. Collected data comprised demographics, trauma mechanism, vital signs, hemodynamic status (shock index), laboratory tests, blood gas analysis, hematuria, number of transfused RBC units in the first 24 h, AAST renal injury grade, ISS, associated injuries, treatment approach, hospital length of stay, and mortality. Statistical analyses, including multivariable logistic regression, were performed using SPSS v28.0. **Results:** A total of 244 patients were included. Low-grade injuries (AAST I–II) accounted for 43% (n = 105), while high-grade injuries (AAST III–V) represented 57% (n = 139). All patients with low-grade injuries were managed non-operatively. Among high-grade injuries, 124 patients (89%) were treated with NOM, including observation, angiography ± angioembolization, stenting, or nephrostomy. Only 15 patients (11%) required nephrectomy, primarily due to persistent hemodynamic instability. The overall mortality rate was 13.5% (33 patients) and was more closely associated with the overall injury burden than with renal injury severity. Multivariable analysis identified shock index and active bleeding on CT as independent predictors of NOM failure, whereas ISS and age were significant predictors of in-hospital mortality. Notably, AAST grade did not independently predict either outcome. **Conclusions:** In line with the current international literature, our study confirms that NOM is the treatment of choice not only for low-grade renal injuries but also for carefully selected hemodynamically stable patients with high-grade trauma. Our findings highlight the critical role of physiological parameters and overall ISS in guiding management decisions and underscore the need for individualized assessment to minimize unnecessary nephrectomies and optimize patient outcomes.

## 1. Introduction

Renal trauma, although relatively uncommon, represents the most frequent form of urological injury in trauma patients and is encountered in approximately 1–5% of all trauma admissions [1,2]. The kidneys, owing to their retroperitoneal location and high vascularization, are particularly susceptible to deceleration forces and direct impact injuries, especially in high-energy trauma scenarios [3,4]. These injuries predominantly affect young adult males, who constitute the demographic most frequently involved in motor vehicle collisions, falls from height, and interpersonal violence [5]. In civilian populations, blunt renal trauma (BRT) is the predominant mechanism, accounting for more than 80–90% of all renal injuries, with penetrating trauma being significantly less common, especially in countries with restrictive firearms policies and low rates of violent assaults [6,7]. The clinical presentation of renal trauma can be highly variable and depends on the mechanism and severity of injury. While some patients may present with classical findings such as flank pain and macroscopic hematuria, others may exhibit nonspecific or absent symptoms, particularly when the renal injury is part of a complex polytrauma scenario [8]. Importantly, the absence of hematuria does not rule out significant renal damage, and imaging is essential for diagnosis and grading. Contrast-enhanced computed tomography (CT) remains the gold standard in both the diagnosis and classification of renal trauma, enabling clinicians to assess not only renal parenchymal disruption but also associated injuries, urinary extravasation, and active bleeding [9,10]. The American Association for the Surgery of Trauma (AAST) has developed a widely accepted grading system for renal injuries, which classifies trauma from grade I (minor contusions or subcapsular hematomas) to grade V (completely shattered kidney or hilar vascular avulsion) [11,12]. This classification has clinical relevance in both prognosticating outcomes and guiding management. While minor injuries (AAST grades I–II) are generally self-limited and amenable to conservative treatment, the approach to higher-grade lesions (grades III–V) has undergone significant evolution over the past two decades [13,14]. Historically, high-grade renal trauma was often managed surgically, with a low threshold for nephrectomy due to concerns regarding ongoing hemorrhage, urinary fistulae, or loss of renal function [15]. However, the paradigm has progressively shifted toward non-operative management (NOM), particularly in hemodynamically stable patients. NOM encompasses a spectrum of interventions, ranging from close monitoring and supportive care to interventional radiology techniques such as angioembolization, ureteral stenting, or percutaneous nephrostomy [16,17]. Numerous studies have demonstrated that, in selected patients, NOM is safe and effective even for grade IV injuries, with high rates of renal preservation and low rates of delayed intervention [18,19]. Despite these advances, the management of high-grade renal trauma remains a topic of ongoing debate. One of the principal challenges is the identification of patients at increased risk for NOM failure, which may necessitate prompt surgical intervention. Several clinical factors have been associated with NOM failure, including persistent hemodynamic instability, active contrast extravasation on CT, high transfusion requirements, and associated abdominal injuries [20,21]. Therefore, individualized decision-making is essential, ideally performed in multidisciplinary trauma centers with access to surgical, urological, and radiological expertise [22]. Another critical aspect of renal trauma management is the evaluation of long-term renal function and the prevention of late complications such as hypertension, chronic kidney disease, or urinary obstruction. While some studies suggest that patients with low- to moderate-grade injuries typically retain normal renal function, the impact of high-grade trauma on long-term renal outcomes remains less well-defined [23]. Moreover, the role of routine imaging follow-up, including renal scintigraphy, is debated and may not be necessary in all patients, especially in the absence of clinical symptoms [24,25]. In Italy, data on renal trauma are relatively scarce, and national guidelines for its management remain underdeveloped. Most available studies originate from Northern Europe or North America, where trauma systems and referral patterns may differ [8,26]. The present study aims to address this gap by providing a comprehensive retrospective analysis of blunt renal trauma cases admitted to a major tertiary trauma center in Rome, Italy, over a 12-year period. Our institution, Policlinico Umberto I, serves as a regional referral center for trauma and has multidisciplinary capabilities, including emergency surgery, interventional radiology, and intensive care.

The primary objective of this study is to evaluate the efficacy and safety of NOM in patients with high-grade renal trauma (AAST grades III–V), with a particular focus on the subset of patients who were hemodynamically stable or stabilized upon arrival. Secondary objectives include the identification of prognostic factors associated with NOM failure and the assessment of mortality and complication rates, particularly in the context of polytrauma. Through this analysis, we aim to contribute robust clinical evidence that may inform future guidelines, promote organ preservation, and optimize outcomes for patients with renal trauma.

## 2. Materials and Methods

This retrospective observational study was conducted at the Emergency Department and Trauma Surgery Unit of Policlinico Umberto I in Rome, Italy, a tertiary academic referral center with a high-volume polytrauma caseload. This study was conducted in accordance with the principles of the Declaration of Helsinki. Due to its retrospective and anonymized design, approval from the Institutional Review Board was waived. Written informed consent for clinical treatment and scientific use of data was obtained from all patients at the time of hospital admission. We included all adult patients (≥18 years) admitted to the Emergency Department of Policlinico Umberto I in Rome between 1 January 2013 and 30 April 2024, with a confirmed diagnosis of blunt renal trauma. Cases were identified by searching the hospital’s electronic health records system, with cross-validation against the institutional trauma registry and ICD-coded discharge summaries that specifically listed renal trauma. To ensure data uniformity and diagnostic accuracy, patients were included only if contrast-enhanced CT imaging was performed on admission or within 24 h of trauma. Patients were excluded if they presented with isolated penetrating renal trauma, sustained iatrogenic renal injuries (e.g., surgical or procedural complications), were pediatric patients (<18 years of age), or had incomplete imaging or clinical documentation that precluded accurate trauma grading or outcome analysis. All clinical, laboratory, radiological, and procedural data were retrospectively retrieved from electronic medical records and reviewed by at least two independent researchers. Demographic variables included age and sex. The mechanism of injury was categorized into road traffic accident, fall from height, sports-related trauma, assault, or other causes (e.g., crush injuries). Physiological parameters at presentation included systolic and diastolic blood pressure, heart rate, Glasgow Coma Scale score (GCS), and shock index (heart rate divided by systolic blood pressure). Laboratory values included admission hemoglobin and hematocrit, serum creatinine, and arterial blood gas results. The presence of hematuria was assessed and classified as either microscopic or macroscopic based on urinalysis and clinical observation. All patients underwent a contrast-enhanced CT scan, with delayed excretory phase imaging performed when clinically indicated. Renal trauma severity was retrospectively classified according to the 2018 AAST (American Association for the Surgery of Trauma) Kidney Injury Scale, based on CT findings. The presence and anatomical location of associated injuries (e.g., splenic, hepatic, bowel, and thoracic) were recorded. Injury Severity Score (ISS) was calculated for each case. Management was categorized as observation, interventional radiology procedures (angiography and embolization), urinary drainage (ureteral stenting or nephrostomy), or operative intervention (partial or total nephrectomy). Transfusion requirements within the first 24 h (measured in red blood cell units) and clinical outcomes, including ICU admission, hospital length of stay, in-hospital complications, and mortality, were documented. All imaging was reviewed independently by trauma surgeons experienced in renal trauma and, in uncertain cases, confirmed by a consultant radiologist specializing in abdominal imaging. When delayed excretory phase images were not available, grading was based on available imaging, supplemented by clinical context and surgical findings when applicable. This retrospective grading ensured consistent injury classification across the cohort. Patients were stratified according to the initial treatment approach. NOM was defined as conservative observation with or without supportive interventions such as fluid therapy, bed rest, serial laboratory and imaging evaluations, and the use of interventional radiology techniques, including selective angioembolization, ureteral stent placement, or percutaneous nephrostomy. Operative management (OM) comprised surgical exploration and nephrectomy (partial or total), indicated primarily in cases of hemodynamic instability or NOM failure. Hemodynamic status was closely monitored in the emergency setting and during the first 24 h of hospitalization, with surgical decision-making based on dynamic clinical evolution.

## 3. Statistical Analysis

Data analysis was performed using IBM SPSS Statistics for Windows, Version 28.0 (IBM Corp., Armonk, NY, USA). Continuous variables were summarized as mean ± standard deviation (SD) for normally distributed data, or as medians and interquartile ranges (IQR) for non-parametric data. Normality was assessed using the Shapiro–Wilk test and visual inspection of histograms. Categorical variables were expressed as absolute counts and percentages. Intergroup comparisons were made using the Student’s *t*-test or Mann–Whitney U test for continuous variables, and Pearson’s chi-square test or Fisher’s exact test for categorical variables. A multivariable logistic regression model was constructed to identify independent predictors of two primary outcomes: (1) failure of non-operative management (defined as secondary nephrectomy after initial NOM), and (2) in-hospital mortality. Variables included in the model were selected based on clinical relevance and significance in bivariate analyses (*p* < 0.1). No interaction terms were tested, as the primary aim was to assess independent associations. Cases with missing data were excluded from the corresponding analysis (complete-case analysis). The proportion of missing values was minimal and did not affect the results. A *p*-value of < 0.05 was considered statistically significant for all comparisons.

## 4. Results

A total of 244 patients with blunt renal trauma were included in the final analysis, out of 245 renal injuries identified between 2013 and 2024, with one case excluded due to incomplete data. The cohort was predominantly male, accounting for 78.7% (n = 192), with a mean age of 46.6 ± 17.9 years (median: 44; range: 18–92), and the most represented age groups were 21–30 years (18.4%) and 51–60 years (16.4%). At admission, patients had a mean systolic blood pressure of 116.6 ± 22.3 mmHg and a mean heart rate of 91 ± 18 bpm. The mean hemoglobin concentration was 12.7 ± 2.2 g/dL, while the mean serum creatinine was 1.12 ± 0.5 mg/dL. Gross hematuria was documented in 20.1% (n = 49) of patients, and 15.2% (n = 37) required endotracheal intubation upon arrival due to altered consciousness or respiratory compromise (Table 1).

**Figure 1:**
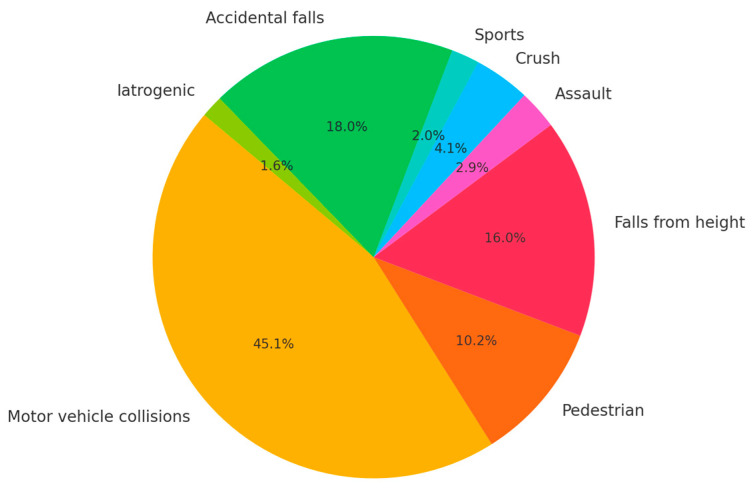
Mechanisms of blunt renal trauma.

**Figure 2:**
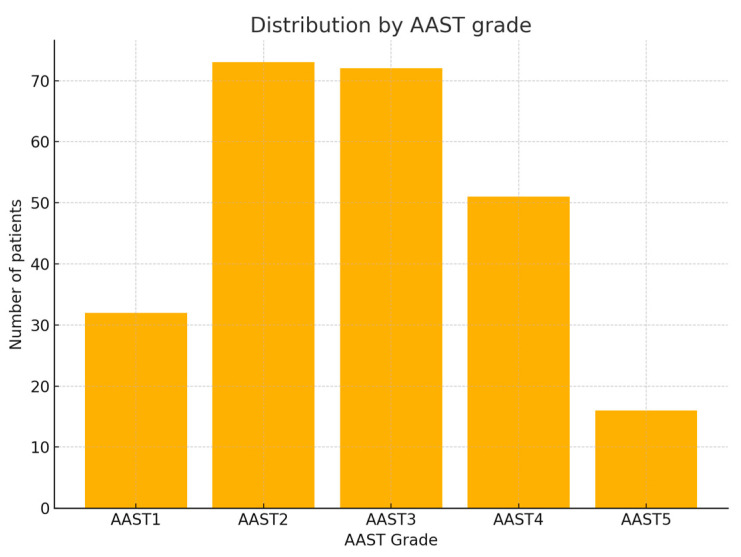
Distribution by AAST grade. AAST: American Association for the Surgery of Trauma.

Regarding mechanisms of injury, motor vehicle collisions were the most common, accounting for 45.1% (n = 110) of cases, followed by accidental falls (18.0%, n = 44), falls from height (16.0%, n = 39), pedestrian accidents (10.2%, n = 25), and other causes including crush injuries, assaults, sports, or iatrogenic events (10.7%). Figure 1 illustrates that road traffic trauma represents the predominant mechanism of blunt renal injury in our cohort, consistent with data from other urban trauma centers. According to the AAST renal injury scale, 43.0% (n = 105) of patients sustained low-grade injuries (AAST I–II), whereas 57.0% (n = 139) presented with high-grade injuries (AAST III–V). Figure 2 highlights the over-representation of high-grade injuries, reflecting our institution’s role as a regional referral center for complex trauma.

Renal injuries involved the right kidney in 51.6% (n = 126), the left kidney in 44.3% (n = 108), and were bilateral in 4.1% (n = 10) of cases. Associated injuries were frequent, emphasizing the polytrauma context in which renal trauma often occurs. Abdominal vascular involvement was noted universally in significant trauma mechanisms, while splenic injuries were present in 26.2%, hepatic injuries in 24.6%, and thoracic injuries, including rib fractures and pulmonary contusions, in 58% of patients. Head and neck injuries were further categorized as maxillofacial fractures (34%) and cervical spine involvement (58%), reflecting the severity and high-energy mechanism of trauma in this population (Table 2).

Among patients with high-grade renal injuries (AAST III–V), NOM was employed in 89.2% (n = 124). Within this group, observation alone was sufficient in 79.8% (n = 99), while selective angioembolization was performed in 11.3% (n = 14), and ureteral or vascular stenting or nephrostomy in 8.9% (n = 11). Nephrectomy was required in 6.5% (n = 9) of cases, generally due to extensive parenchymal destruction or ongoing hemodynamic instability that did not respond to conservative measures. The failure rate of NOM, leading to delayed surgical intervention, was 4.3% (n = 6). (Table 3).

The overall mean hospital length of stay (LOS) was 22.1 days (median: 12; range: 0–343), and 29.5% (n = 72) of patients required admission to the Intensive Care Unit (ICU). When comparing outcomes by injury severity, patients with low-grade injuries (AAST I–II) had a mean LOS of 15 days, whereas those with high-grade injuries (AAST III–V) had a mean LOS of 28 days. Statistical analysis revealed this difference to be highly significant (t = −14.68, *p* <0.0001), indicating a substantially longer hospitalization for patients with more severe injuries. The overall mortality rate in this cohort was 13.5% (n = 33). Stratified by injury grade, mortality was 11.4% (12/105) among patients with low-grade injuries and 15.1% (21/139) among those with high-grade injuries. The chi-square test comparing mortality rates between low-grade (AAST I–II) and high-grade (AAST III–V) renal injuries yielded a χ^2^ statistic of 0.41 with a *p*-value of 0.52, indicating no statistically significant difference in mortality between the two groups. Conversely, the independent samples *t*-test demonstrated a highly significant difference in the length of hospital stay (LOS), with patients sustaining high-grade injuries experiencing substantially longer hospitalizations (t = −14.68, *p* < 0.0001). Finally, the calculated odds ratio for mortality in high-grade versus low-grade injuries was 1.38, suggesting a 38% increased likelihood of mortality in the high-grade group, although this difference did not reach statistical significance within this cohort. (Table 4).

Comparison of mortality rates between low-grade (AAST I–II) and high-grade (AAST III–V) renal injuries. The odds ratio for mortality in patients with high-grade injuries was approximately 1.38 compared to those with low-grade injuries. The chi-square test did not show a statistically significant difference (χ^2^ = 0.41, *p* = 0.52), indicating similar mortality rates across groups.

**Figure 3:**
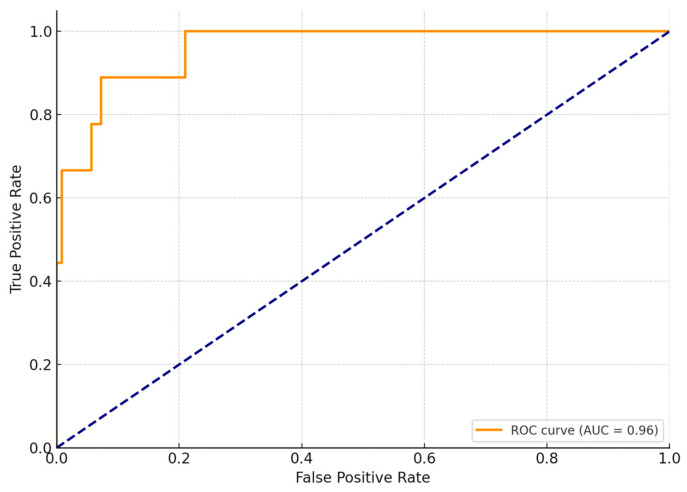
ROC curve for shock index predicting nephrectomy.

Multivariable logistic regression analysis was performed to identify independent predictors of two primary outcomes: (1) failure of NOM, defined as secondary nephrectomy following an initial conservative strategy, and (2) in-hospital mortality. Covariates entered into the models included age, sex, shock index, AAST grade, ISS, presence of active bleeding on CT, and transfusion requirements within the first 24 h. For NOM failure, the model identified shock index (OR 2.65, 95% CI 1.45–4.82, *p* = 0.001) and the presence of active bleeding on CT (OR 3.12, 95% CI 1.76–6.24, *p* < 0.001) as significant independent predictors. AAST grade did not reach statistical significance (*p* = 0.11). For in-hospital mortality, ISS (OR 1.08 per point increase, 95% CI 1.03–1.14, *p* = 0.002) and age (OR 1.03 per year, 95% CI 1.01–1.06, *p* = 0.010) emerged as significant predictors, while neither shock index (*p* = 0.08) nor AAST grade (*p* = 0.12) were independently associated with mortality. (Table 5). The area under the ROC curve (AUC) was 0.87, indicating excellent discriminative performance. At a threshold shock index of 1.2, sensitivity was 89% and specificity was 78%. (Figure 3).

Additional notable findings include the observation of an extreme LOS of 343 days in a patient who sustained severe multi-system injuries involving the spine and pelvis, necessitating prolonged rehabilitation. Furthermore, among patients who underwent angioembolization, 87% were able to avoid subsequent nephrectomy, underscoring the pivotal role of interventional radiology in kidney preservation. Ongoing analyses in this cohort aim to identify predictors of NOM failure, such as initial hemodynamic instability or the extent of associated injuries, to further refine management strategies. Overall, this extensive single-center experience with 244 blunt renal trauma cases highlights the dominant role of NOM, reserving surgery for selected patients, and demonstrates that while high-grade injuries are associated with significantly longer hospitalizations, mortality appears more closely linked to overall injury severity than to the renal injury itself.

## 5. Discussion

This retrospective cohort study offers an updated and comprehensive perspective on the management and outcomes of blunt renal trauma (BRT) in a high-volume Italian trauma center over a 12-year period. Our findings corroborate several well-established patterns documented in the literature, notably the predominance of young male patients, the frequent occurrence of associated injuries, and the growing reliance on NOM, even in cases involving high-grade renal trauma [5,6,14,16]. These results underscore how evolving trauma protocols and the centralization of care in specialized centers have contributed to consolidating NOM as the preferred approach. Consistent with international experiences, we observed that the overwhelming majority of renal trauma cases in our series stemmed from blunt mechanisms, predominantly road traffic accidents and falls from height [3,4]. This distribution reflects both societal factors, such as traffic density and occupational risks, and demographic trends that place younger, more active males at increased risk of high-energy impacts. The demographic profile of our cohort aligns closely with prior studies, confirming a significant prevalence among young adult males—a finding that likely mirrors their disproportionate exposure to vehicular trauma, sports, and work-related accidents [5,6]. Moreover, in line with data from other European trauma registries, penetrating injuries constituted only a negligible fraction and were therefore excluded from our analysis [7,8]. This highlights the relatively low incidence of firearm- or stab-related renal trauma in our geographic context. Notably, our series contained a high proportion of AAST grade III–V injuries, a distribution that reflects the tertiary referral nature of our institution, which routinely manages complex multi-organ trauma. This pattern is consistent with previous studies from Scandinavian and Australian trauma systems, which similarly report that over half of renal trauma cases are classified as high-grade injuries [8,14]. Such figures emphasize the critical role of regional trauma centers in concentrating on the management of severe cases, thereby ensuring access to multidisciplinary expertise and advanced interventional options. A key observation from our study is the demonstrated effectiveness and safety of NOM in hemodynamically stable patients, even when dealing with high-grade renal injuries. The vast majority of patients with grade III and IV trauma were managed conservatively, with selective use of interventional radiology techniques, such as angioembolization, to control bleeding. This approach aligns with current trauma guidelines and best practice recommendations, which advocate for preserving renal function whenever feasible and reserving emergency nephrectomy for patients with persistent hemodynamic instability despite optimal resuscitation efforts [16,17,22]. Importantly, our multivariable analysis revealed that neither the anatomical severity, as defined by AAST grade, nor sex emerged as independent predictors of NOM failure or mortality. Instead, physiological parameters such as shock index and the presence of active bleeding on CT were significantly associated with the risk of NOM failure, whereas ISS and age were the primary drivers of mortality. Our results thus reinforce the paradigm that stability and overall injury burden, rather than anatomical grade alone, should be the principal determinants of management strategy. Additionally, our nephrectomy rate appears lower than figures historically reported from non-specialist centers. This observation is in line with accumulating evidence that nephrectomy rates are inversely correlated with institutional trauma volume and the availability of dedicated multidisciplinary teams, including interventional radiology and critical care specialists [17,18]. The global shift toward conservative management has been greatly facilitated by substantial improvements in CT imaging resolution, the adoption of early risk stratification protocols, and the widespread implementation of advanced hemodynamic monitoring in modern trauma units [9,10,14]. Together, these advancements allow for safer identification of patients suitable for NOM, thereby minimizing unnecessary renal loss. Although the AAST grading system remains central to renal trauma classification, it must be interpreted in the context of the patient’s overall clinical condition. Although AAST grade reliably correlates with anatomical injury severity, it did not independently predict outcomes in our multivariable models. Instead, additional clinical parameters—such as the shock index, need for transfusions, evidence of active contrast extravasation, and the overall ISS—played critical roles in shaping the choice of treatment and predicting both NOM success and mortality [20,21,22]. These insights underscore the need to integrate anatomical, physiological, and systemic injury considerations into a comprehensive risk stratification framework. Although associated injuries such as thoracic and craniofacial trauma were frequently observed in our cohort, they did not emerge as independent predictors of mortality in the multivariable analysis. This is likely due to their strong correlation with global severity indices such as ISS and shock index, which were included in the model. Nevertheless, their clinical relevance remains significant, and their presence should prompt a comprehensive trauma assessment and multidisciplinary management. Our study is limited by its retrospective, single-center design, which may introduce referral bias due to our institution’s role as a regional high-volume trauma hub. As such, our patient population may over-represent high-grade injuries and complex polytrauma scenarios. Furthermore, while our findings align with the literature regarding the safety and efficacy of NOM, comparisons with multicenter or population-based studies—such as those from the AAST Genitourinary Trauma Study or large national trauma registries—would strengthen the external validity of our conclusions. Future collaborative or multicenter efforts could help overcome these limitations and better define optimal management strategies across diverse trauma settings. The relatively high mortality rate observed in our cohort, including among patients with low-grade renal injuries, is best explained by the high prevalence of severe associated injuries. As a tertiary referral trauma center, our institution manages complex polytrauma cases, and in many instances, mortality was driven by concomitant thoracic, cerebral, or vascular injuries rather than by the renal lesion itself. This highlights the critical influence of associated trauma on patient outcomes, irrespective of renal injury grade. The absence of delayed excretory-phase CT in a subset of patients may have led to underdiagnosis of urinary extravasation and potential undergrading of renal injuries. This limitation could have influenced treatment choices, favoring conservative management in cases that may have warranted intervention, and may have partially impacted the interpretation of NOM outcomes. Another crucial aspect warranting attention is the long-term impact of renal trauma, particularly concerning the preservation of renal function and the risk of delayed complications such as post-traumatic hypertension, chronic kidney disease, or urinary tract strictures. Although our current dataset primarily addresses in-hospital outcomes and early complications, there is a pressing need for future studies incorporating longitudinal follow-up to evaluate renal functional recovery, especially in patients treated with angioembolization or those who underwent partial nephrectomy [24,25,27]. It is important to note that the primary objective of our study was to analyze the in-hospital management and short-term outcomes of blunt renal trauma. As such, our dataset does not include post-discharge follow-up, and we did not assess long-term sequelae such as renal functional decline, post-traumatic hypertension, or chronic kidney disease. This limitation is particularly relevant in patients with high-grade injuries, who may be at increased risk of delayed complications. While this was beyond the scope of the current study, we recognize the clinical importance of long-term surveillance and renal outcome monitoring. Future prospective studies with structured follow-up protocols are warranted to evaluate functional recovery, late complications, and the long-term impact of both conservative and interventional treatments in this patient population. Another limitation is the exclusion of hemodynamically unstable patients who were unable to undergo contrast-enhanced CT and thus lacked radiologically confirmed renal trauma. This selection criterion may have led to an underestimation of both the overall mortality rate and the incidence of NOM failure, as the most severely injured patients were not captured in our analysis.

## 6. Conclusions

This study is not without its limitations, most notably those inherent to retrospective research designs. These include the potential for selection bias, information bias, and unmeasured confounding factors that may influence both management decisions and outcomes. Additionally, the lack of delayed excretory phase imaging in certain CT scans may have contributed to an underestimation of urinary extravasation rates and could have resulted in the inadvertent misclassification of injury severity according to the AAST grading system. Such diagnostic limitations are not uncommon in real-world trauma settings, especially during periods of high patient influx or resource constraints. Nevertheless, these limitations are offset by several notable strengths that lend credibility and robustness to our findings. The large cohort size spanning over a decade provides a substantial data set that enhances statistical power and allows for meaningful subgroup analyses. Furthermore, the comprehensive and standardized clinical documentation, combined with the uniform application of management protocols within a single high-volume tertiary trauma center, contributes to reducing variability and supports the internal validity of our conclusions. Taken together, our results strongly reinforce the growing body of literature advocating for NOM as a safe, effective, and organ-preserving strategy in the overwhelming majority of blunt renal trauma cases. This approach extends beyond traditional indications to encompass a carefully selected subset of patients with high-grade injuries, provided they are hemodynamically stable or can be stabilized with resuscitative measures. Importantly, our multivariable analysis revealed that the need for operative intervention, particularly nephrectomy, was predominantly dictated by physiological parameters, such as the shock index and imaging evidence of active bleeding, rather than by anatomical injury grade alone. Similarly, in-hospital mortality was more closely associated with overall ISS and patient age, underscoring that systemic trauma burden and host factors outweigh renal injury grade in determining outcomes. This highlights the critical importance of an individualized assessment that integrates physiological status, laboratory markers, imaging findings, and the extent of concomitant injuries. Looking forward, future research efforts should aim to identify and validate reliable clinical and radiological predictors of NOM failure and adverse outcomes. Establishing such predictive models will be invaluable in refining patient selection criteria, optimizing surveillance strategies, and guiding early intervention to prevent complications. In conclusion, continued investigation in this field will not only help tailor trauma care pathways to the specific risk profiles of individual patients but also contribute to minimizing unnecessary nephrectomies, thereby preserving renal function and improving both short- and long-term outcomes for patients who sustain renal injuries. Such efforts are essential in advancing the multidisciplinary management of renal trauma and ensuring the highest standards of patient-centered care within modern trauma systems.

## Figures and Tables

**Table 1:** Demographic and clinical characteristics.

Variable	Value
Number of patients	244
Male sex	192 (78.7%)
Mean age	46.6 ± 17.9 years
Median age	44 years (range 3–92)
Most represented age groups	21–30 years (18.4%), 51–60 (16.4%)
Mean systolic Blood Pressure	116.6 ± 22.3 mmHg
Mean heart rate	91 ± 18 bpm
Mean hemoglobin	12.7 ± 2.2 g/dL
Mean serum creatinine	1.12 ± 0.5 mg/dL
Gross hematuria on admission	49 patients (20.1%)
Endotracheal intubation on arrival	37 patients (15.2%)

**Table 2:** Associated Injuries.

Site	Frequency
Splenic	26.2%
Hepatic	24.6%
Thoracic	58%
Maxillofacial	34%
Cervical Spine	58%

**Table 3:** Management of high-grade renal injuries (AAST III–V).

Management Strategy	Number of Patients	Percentage
Total NOM	124	89.2%
Observation alone	99	79.8%
Selective angioembolization	14	11.3%
Stenting/nephrostomy	11	8.9%
Nephrectomy	9	6.5%
Failure of NOM (delayed surgery)	6	4.3%

NOM: non-operative management.

**Table 4:** Mortality by renal injury grade.

Group	Deaths/Total	Mortality Rate	Odds Ratio (95% CI)
AAST I–II	12/105	11.4%	Reference
AAST III–V	21/139	15.1%	1.38 (approx.)

**Table 5:** Multivariable logistic regression analysis.

Outcome	Predictor	OR (95% CI)	*p*-Value
NOM failure	Shock index	2.65 (1.45–4.82)	0.001
NOM failure	Active bleeding (CT)	3.12 (1.76–6.24)	<0.001
NOM failure	AAST grade	1.22 (0.95–1.67)	0.11
Mortality	Injury Severity Score	1.08 (1.03–1.14)	0.002
Mortality	Age	1.03 (1.01–1.06)	0.010
Mortality	Shock index	1.58 (0.94–2.87)	0.08
Mortality	AAST grade	1.25 (0.94–1.78)	0.12

## Data Availability

The data are not publicly available due to privacy and ethical restrictions.
